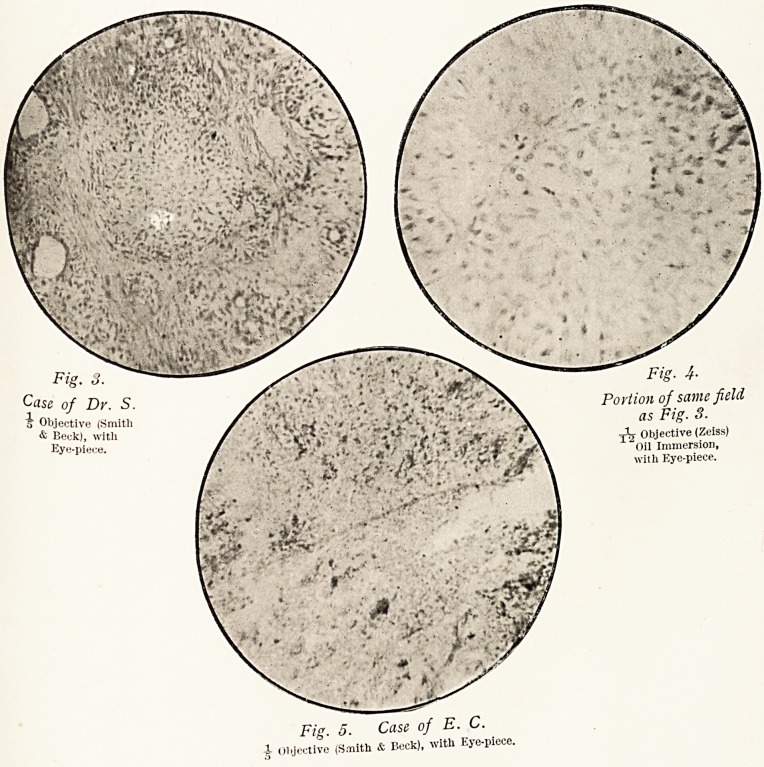# Buccal Glandular Tumours

**Published:** 1895-06

**Authors:** James Swain

**Affiliations:** Assistant-Surgeon to the Bristol Royal Infirmary


### BUCCAL GLANDULAR TUMOURS.

BY

James Swain, M.D., M.S. Lond., F.R.C.S. Eng.,

*Assistant-Surgeon to the Bristol Royal Infirmary.*

---

Solid tumours of the lips and cheek are far from common, and by reason of their peculiar anatomical structure have a greater pathological than clinical interest.

Of two cases which have recently come under my notice, one occurred on the left side of the upper lip of Dr. S., who had complained of it for four years; and the other was in the left cheek, about an inch from the angle of the mouth, of a lad, E. C., twelve years of age, who had noticed the "lump" about two years. In each case relief was sought because of the inconvenience in mastication, the growth being apt to push the mucous membrane between the teeth and so causing it to become nipped. In each case the mucous membrane was slightly fixed to a smooth hard ovoid tumour, about three-quarters of an inch in the long and half-an-inch in the transverse diameter, with some flattening on the side which pressed against the teeth. They were movable in the surrounding tissues; and although in consequence of their size they had separated the muscular fibres of the lip or cheek, they scarcely reached to the subcutaneous connective tissue, and the skin was therefore freely movable over each tumour.

They were removed by incising the mucous membrane over them—in one case, E. C., after the administration of chloroform; in the other, Dr. S., after the injection of half a grain of cocaine into the submucous tissues around the tumour. On fixing a tenaculum into the growth, it was found to "shell out" easily with a few touches of the scalpel or scissors. The edges of the incision were brought together with catgut.

Both tumours were found to be encapsuled, and cut with a rather firm section. One case, Dr. S., presented a yellowish-white irregular surface resembling in form the tops of "granulations" or papillæ; the other, E. C., was somewhat smoother. Histologically the two tumours were different, and must be described separately.

In the case of Dr. S. the growth contained numerous cystic spaces, lined by epithelium and supported by a peculiar form of connective tissue. (FIG. 1.) The spaces, which formed much the smaller portion of the tumour, varied much in size and shape, some of the medium-sized ones being filled with a translucent (mucoid?) material, and the smallest of all looking like rings of epithelium with scarcely any appreciable cavity. Under a higher power (FIG. 2) this epithelium is seen to consist practically of two layers; a deeper one composed of irregularly columnar cells placed side by side, with large and sharply-defined nuclei, and a more superficial layer (nearer the spaces) surmounting these and consisting of a flattened or stratified epithelium. These flattened cells—as in mucous membranes where such cells exist—are distinctly nucleated, and the general appearance of the epithelium surrounding the spaces resembles that of the transitional epithelium of the bladder; but in the case of the tumour the change from columnar to flattened cells is more abrupt, for as a rule there is only a single layer o each.

The interstitial tissue (FIG. 3) is mainly cellular; but here and there are strands of fibrous tissue, containing only a few cells, and patches which appear to be of a myxomatous nature. The cells of this supporting tissue present, under a high power (FIG. 4), a great diversity of form, being angular, oat-shaped and round, and looking much like sarcomatous tissue. In some places the cells are found branched, and in others lengthening out into fibres.

The other tumour, case of E. C., is composed of large round nucleated cells (FIG. 5) supported by a retiform tissue, and resembles the structure of a lymphatic gland, except that some of the cells are larger and the retiform tissue is less distinct. Bands of fibrous tissue and capillary vessels are occasionally seen. Unfortunately only a few sections of this tumour were cut, so that I am unable to say whether the structure was the same throughout the growth.

Before drawing any conclusion as to the origin of these tumours, it will be well to consider briefly the records of other cases.

Mr. (afterwards Sir W.) Lawrence reports,[1] in his observations on tumours, the removal of one of the size of a walnut from the upper lip of a young lady aged nineteen years. The growth was movable, the surface "knotted," and its substance was "whitish, compact, tough, and almost of cartilaginous firmness." It contained also a little "bony matter" at one point.

Sir James Paget refers[2] to a growth of twelve years' duration removed by Mr. Lloyd from the upper lip of a healthy man. On section the tumour was firm, slightly lobed, and in general aspect like a parotid tumour, but histologically was a perfect imitation of lobulated gland structure as found in the breast.

Paget himself[3] removed a similar tumour from the upper lip of a man aged thirty, which had been growing for four years. It was less distinctly glandular than the foregoing, and contained a portion of bone in the centre, as in Lawrence's case

[1] *Med.-Chir. Tr.*, vol. xvii., 1832, p. 28.
[2] *Lectures on Tumours*, 1853, p. 262. [3] *Op. cit.*, p. 263.

*Fig. 1. Case of Dr. S.*

$\frac{1}{5}$ Objective (Smith & Beck), with Eye-piece.

*Fig. 2. Portion of same field as Fig. 1.*
$\frac{1}{12}$ Objective (Zeiss) Oil Immersion.

*Fig. 3.*

*Case of Dr. S.*

$\frac{1}{5}$ Objective (Smith & Beck), with Eye-piece.

*Fig. 4.*
*Portion of same field as Fig. 3.*
$\frac{1}{12}$ Objective (Zeiss) Oil Immersion, with Eye-piece.

*Fig. 5. Case of E. C.*

$\frac{1}{5}$ Objective (Smith & Beck), with Eye-piece.

(*vide supra*), to which Sir James Paget refers in a later edition[1] of his work.

Billroth[2] removed a tumour one inch from the angle of the mouth, which twice recurred.

Francis Mason[3] removed a growth the size of a Tangerine orange from the left side of the upper lip of a woman aged forty-five. The tumour was supposed to have been caused by a blow on the lip at four years of age, and had been growing slowly ever since. It was a firm elastic lobulated growth of a glandular character, containing a few delicate calcareous plates.

Prof. Humphry[4] removed a similar tumour from the left side of the upper lip of a woman aged thirty-five, which contained hyaline cartilaginous areas and numerous tracts of glandular structure "undergoing a variety of complex transformation;" and Goodhart[5] had two cases which contained cartilaginous, osseous, myxomatous, and yellow elastic tissue but with scarcely any glandular tissue.

In reading these various accounts one is struck with the diversity and complexity of the structure presented by these tumours—circumstances which, I think, point to their probable embryonic origin.

In the case of Dr. S., the interstitial portion is of an embryonic type, and quite unlike any adult form of connective tissue; and, granting for the moment their embryonic origin, I think we have a sufficient explanation for their subsequent conversion into the myxomatous, cartilaginous, and other forms of connective tissue. The fact that this interstitial portion of Dr. S.'s tumour resembled sarcomatous tissue (itself also of an embryonic type) must not be supposed to indicate a malignant nature; for the encapsulation, duration, and clinical aspect of these growths attest their benign character. So too the fact that some of these recorded tumours have recurred after removal is no evidence, *per se*, of their malignancy.

[1] *Lectures on Surgical Pathology*, edited by William Turner, 4th Ed., 1876, p. 564.
[2] *Virchow's Archiv.*, bd. xvii., p. 374, quoted by Sir James Paget on p. 566.
[3] *Brit. M. J.*, 1868, vol. ii., p. 389.
[4] *Ibid*, 1880., vol. i., p. 816. [5] *Lancet*, 1876,. vol. ii., p. 574.

The tumour in the case of E. C., being apparently composed of lymphoid tissue only, is unlike the other described cases; but on the "embryonic theory" we might regard it as the conversion of a growth of embryonic connective tissue into the lymphoid variety, corresponding to the myxomatous and other varieties of connective tissue in the other tumours. As already stated, only a few sections of this growth were obtained, but it is peculiar that lymphoid tissue does not appear to have been found in any of the other cases. Some colour is, however, lent to the argument here adduced by a case of tumour of the palate [1] (and these growths appear to me to closely resemble[2] those which form the subject of this paper) described by Jonathan Hutchinson, in which glandular acini and lymph follicles were found lying side by side.

Turning to the glandular (cystic) portion, we find that the epithelium lining these cavities, as I have described it in the case of Dr. S., is very like the lining epithelium of the ducts of the mucous glands of the mouth. Of the latter, Klein and Noble Smith say[3]: "The duct possesses a large lumen, and this is in man lined with a single layer of beautiful columnar epithelial cells, each with an oval nucleus . . . At the mouth the stratified epithelium of the surface passes, in an attenuated form, a short distance into the duct, but is soon replaced by the above columnar epithelium." Now, the epithelial lining of the mouth is derived embryologically from an infolding of the epiblast, and the buccal glands are formed from this again by recession or inversion so as to form mucous follicles. It is precisely this same form of epithelium which we find lining the cystic spaces of these tumours, and it is easy to understand how this process of inversion during the formation of the mucous crypts might become faulty, and leave a portion of embryonic glandular and connective tissue shut off from the ordinary mucous surface. Indeed, faulty developments of the mouth are amongst the commonest of teratological errors, and it is remarkable that all these tumours (Goodhart gives no account

[1] *Tr. Path. Soc. Lond.*, vol. xxxvii., 1886, p. 490.

[2] See a paper by Stephen Paget on "Tumours of the Palate," in *Tr. Path. Soc. Lond.*, vol. xxxviii., 1887, p. 348.

[3] *Atlas of Histology*, 1880, p. 195.

of the position of his) have occurred in or near the *upper* lip, which is also the usual seat of labial deformity.

The portion of embryonic tissue thus isolated may remain dormant until some irritation (from a blow, teeth, etc.) rouses it to a more active growth. The lip, from its exposed position and mobility, would be a likely place for such irritation to occur, and this may be one of the factors in the late growth of these tumours.

The apparent predilection for the *left* side in the cases here recorded is remarkable but inexplicable.

I must express my indebtedness to Mr. James Taylor for his great kindness and skill in the production of the photographs with which this paper is illustrated.

## Figures and Tables

**Fig. 1. f1:**
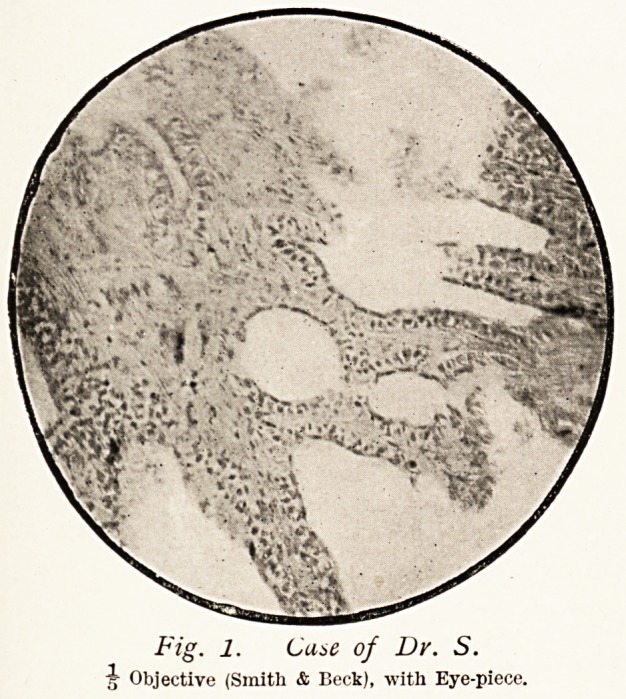


**Fig. 2. f2:**
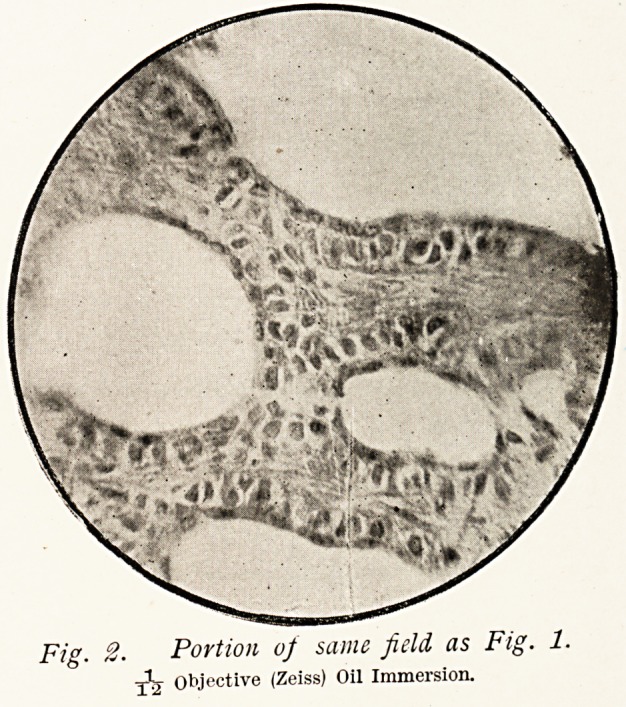


**Fig. 3. Fig. 4. Fig. 5. f3:**